# The last and first frontier – emerging challenges for HIV treatment and prevention in the first week of life with emphasis on premature and low birth weight infants

**DOI:** 10.7448/IAS.18.7.20271

**Published:** 2015-12-02

**Authors:** Mark F Cotton, Sandi Holgate, Aurelie Nelson, Helena Rabie, Catherine Wedderburn, Mark Mirochnick

**Affiliations:** 1 Division of Paediatric Infectious Diseases, Department of Paediatrics and Child Health, Faculty of Medicine and Health Sciences, Tygerberg Children's Hospital, Stellenbosch University, Stellenbosch, South Africa; 2 Division of Neonatology, Department of Paediatrics and Child Health, Faculty of Medicine and Health Sciences, Tygerberg Children's Hospital, Stellenbosch University, Stellenbosch, South Africa; 3 Médecins Sans Frontières, Khayelitsha, South Africa; 4 Department of Pediatrics, Boston University School of Medicine, Boston, MA, USA

**Keywords:** premature, low birth weight, small for gestation, antiretrovirals, management

## Abstract

**Introduction:**

There is new emphasis on identifying and treating HIV in the first days of life and also an appreciation that low birth weight (LBW) and preterm delivery (PTD) frequently accompany HIV-related pregnancy. Even in the absence of HIV, PTD and LBW contribute substantially to neonatal and infant mortality. HIV-exposed and -infected infants with these characteristics have received little attention thus far. As HIV programs expand to meet the 90-90-90 target for ending the HIV pandemic, attention should focus on newborn infants, including those delivered preterm or of LBW.

**Discussion:**

In high prevalence settings, infant diagnosis of HIV is usually undertaken after the neonatal period. However, as *in utero* infection may be diagnosed at birth, earlier initiation of therapy may limit viral replication and prevent early damage. Globally, there is growing awareness that preterm and LBW infants constitute a substantial proportion of births each year. Preterm infants are at high risk for vertical transmission. Feeding difficulties, apnoea of prematurity and vulnerability to sepsis occur commonly. Feeding intolerance, a frequent occurrence, may compromise oral administration of medications. Although there is growing experience with post-exposure prophylaxis for HIV-exposed term newborn infants, there is less experience with preterm and LBW infants. For treatment, there are even fewer options for preterm infants. Only zidovudine has adequate dosing recommendations for treating term and preterm infants and has an intravenous formulation, essential if feeding intolerance occurs. Nevirapine dosing for prevention, but not treatment, is well established for both term and preterm infants.

HIV diagnosis at birth is likely to be extremely stressful for new parents, more so if caring for preterm or LBW infants. Programs need to adapt to support the medical and emotional needs of young infants and their parents, where interventions may be lifesaving.

**Conclusions:**

New focus is required for the newborn baby, including those born preterm, with LBW or small for gestational age to consolidate gains already made in early diagnosis and treatment of young children.

## Introduction

The Joint United Nations Program on HIV/AIDS (UNAIDS) was established in 1996 to lead global efforts for universal access to prevention, treatment, care and support [1]. There has been much progress, with increased access to care, reduction in vertical transmission and reversal of mortality trends. In 2014, UNAIDS released the 90-90-90 policy document, which defines new global targets to consolidate gains and ultimately end the pandemic. The aims are as follows: identifying HIV in 90% of the infected population, of whom 90% should be placed on combination antiretroviral therapy (cART), of whom 90% should be virally suppressed [2].

Newborn infants are an important target group that includes the most neglected population of all – those with low birth weight (LBW) and/or preterm delivery (PTD). Collectively, these infants are the last frontier for HIV management in terms of knowledge and experience. Paradoxically, as they are developmentally the youngest and most fragile population, they also represent the first frontier to be encountered. We comment on the current state of knowledge, areas requiring increased attention under the present circumstances and research. We also seek to heighten awareness in healthcare workers of challenges and opportunities in addressing the needs of young newborn infants, including PTD and LBW infants.

## Discussion

### HIV infection and the newborn infant

Early infant diagnosis leading to prompt initiation of effective cART can prevent postnatal HIV disease progression. Until recently, infant diagnosis focused on those between four and six weeks of age after preliminary data from the Children with HIV Early Antiretroviral (CHER) trial results were released in 2007. This study showed that commencing antiretroviral therapy (ART) at a median of 7.4 weeks of age reduced mortality by 76% and HIV disease progression by 75% in infants with a baseline CD4% ≥25%. However, less well appreciated was data suggesting rapid disease progression preceding ART initiation in the trial. Over the three to four weeks between diagnosis and study entry, seven HIV-infected infants (1.25% of those identified) had already died, 16 (2.8%) had developed signs of advanced HIV disease and almost 20% had a CD4% <25% [3]. These observations, during this short period, suggest that seven weeks of age for ART initiation is too late for many. In a subsequent study from the two CHER trial sites between 2007 and 2010, 62% of 250 infants already had advanced HIV disease when ART was initiated by 12 weeks of age, again illustrating rapid disease progression in early infancy [4]. Impetus for a birth PCR (in addition to a PCR later on) is the awareness that most *in utero* infection can be detected on the first day of life, which could allow earlier linkage to care and less attrition [5].

Infants most at risk for acquiring HIV and who could benefit from both enhanced prophylaxis and early diagnosis are those whose mothers either seroconverted late in pregnancy or had not attained viral suppression on ART. Under these circumstances, there is both a need for early diagnosis to establish infection status as soon as possible after birth and to establish enhanced post-exposure prophylaxis for the infants. These numbers can be substantial. For example, in South Africa, where a rapid antibody test is repeated at week 32 and in labour in pregnant women who initially tested negative, 3.3% of mothers had acute seroconversion, accounting for 26% of vertically infected infants in a public program [6]. PTD and being small for gestational age (SGA) are risk factors for intrapartum HIV transmission, probably because of shorter exposure to antenatal cART and more immature mucosal barriers [7–9].

For infants whose mothers were diagnosed with HIV late in pregnancy, the best possible post-exposure prophylaxis is required, accompanied by caregiver counselling on prophylaxis administration to avoid medication errors. A prophylaxis regimen comprising zidovudine plus three doses of nevirapine is now recommended for infants at high risk for vertical transmission [10,11]. It is also essential to continue prophylaxis in infants whose mothers may be failing ART. In many settings, it is standard practice to introduce cART as post-exposure prophylaxis when there is a risk for vertical transmission [12,13]. Although there is no evidence of enhanced prevention, using cART for prevention will then continue, should baseline tests confirm infection.

**Table 1:** ARVs available for term infants in first four weeks of life and for preterm newborn infants

	Term	Preterm	Comments
Reverse transcriptase inhibitors			
Zidovudine	X	X	IV formulation available
Lamivudine	X		Dosage available for first month
Emtricitabine	X		Dosage from 0 to 3 months
Stavudine	X		
Non-nucleoside reverse transcriptase inhibitors			
Nevirapine	X	X	Dosages available for prevention only
Protease inhibitors			
Lopinavir-ritonavir	X (Recommended only after 2 weeks of age and 42 weeks post-conception)		If used earlier than 42 weeks post-conception age, therapeutic drug monitoring and clinical and cardiac monitoring recommended

From Ref. [11].

Early cART initiation within the first few hours may have benefits beyond preventing HIV disease complications. The report of the Mississippi baby who spent almost two years without detectable plasma viremia after starting cART at 31 hours of age suggests that early therapy could limit HIV reservoir size, which may have a dramatic impact on the lifelong course of HIV infection [14,15]. The experience with the Mississippi baby has increased motivation for a birth PCR and cART for both prophylaxis and treatment in newborn infants [15].

#### Low birth weight, premature and SGA infants

LBW is defined as a birth weight below 2500 g. Its global prevalence is 15.5%, accounting for about 20 million infants born each year, 96.5% of them in developing countries [16]. Contributing factors are prematurity, defined as birth before 37 completed weeks of gestation, and being SGA, defined as a birth weight below the 10th centile for gestational age. PTD accounted for 11.1% or 14.9 million babies in 2010 and is the second most common cause of mortality below five years of age, after pneumonia. Most PTDs occur after 32 weeks of gestation [17]. In a survey from East Africa comprising more than 5000 live births, 9.2% were LBW and 4% were PTD. Compared to term delivery, gestational age below 34 weeks gestation had a 58-fold higher death rate. Risk of death was threefold higher for gestational age between 34 and 36 weeks, but if also SGA mortality was 20-fold higher [18]. In a recent South African report of neonates requiring intensive care in Pretoria, 68% were LBW, with 8% weighing below 1000 g, 24% between 1000 and 1499 g, and 24% between 1500 and 2000 g [19]. In an audit of HIV-exposed preterm infants at a tertiary neonatal unit in Cape Town between 2010 and 2011, 3.3% had a birth weight below 1000 g and were significantly more HIV exposed than those with a higher birth weight. Of 51 HIV-exposed infants, mean birth weight was 834 g and mean gestational age was 28.3 weeks. Most were born by caesarean section before active labour, with a transmission rate of 2.7% by six weeks of age [20].

PTD and LBW rates are increasing every year [21]. In 2010, 59% of SGA newborn infants were born at term and 41% were preterm [22]. Infectious causes of SGA include maternal malaria, varicella and syphilis [23]. Maternal tuberculosis, prevalent in many settings where HIV is common, contributes substantially to prematurity and LBW [24,25]. Placental causes include abruption and infarcts; other maternal causes include hypertension, diabetes or exposure to tobacco, alcohol or recreational drugs [23]. HIV infection and ART contribute to LBW, PTD and SGA [26].

Infant consequences of PTD include hypothermia, hypoglycaemia, respiratory distress, apnoea, intraventricular haemorrhage and sepsis and may complicate HIV diagnosis and treatment. Very premature infants may require parenteral nutrition and tube feeding is necessary below around 34 weeks gestation. Gastro-oesophageal reflux occurs commonly putting the infant at risk for apnoea and aspiration [27]. Necrotizing enterocolitis (NEC), a potentially lethal complication often necessitating periods without enteral feeding, is more common in HIV-infected premature infants [28]. Breastfeeding is especially important to prevent NEC [29]. SGA infants have additional risks of polycythaemia and neurological dysfunction related to hypoxia [30].

#### Antiretrovirals for neonates

Neonates have significant differences in physiology that impact drug disposition, so that neonatal drug absorption, distribution, metabolism and elimination differ from that in older infants and children. Neonatal differences in drug disposition are even greater in premature infants [31]. Antiretrovirals (ARVs) cannot be safely and effectively used in neonates without being directly studied in these most vulnerable populations. Immaturity in gastrointestinal tract function, the unique diet of the young infant, developmental changes in drug transporter systems and the need for special formulations due to the inability to swallow pills all impact drug absorption. Maturational changes in body size and composition, as well as plasma protein concentrations, will change drug distribution with age [32]. Activity of enzymes critical for drug elimination such as CYP3A4 and UGT2B6 are lower in neonates than older infants and lower in preterm than term infants. The pattern of maturation in activity is specific for each enzyme and isoform, with adult values generally reached by later childhood or early adolescence [31,32].

Although cART regimens should be an essential component of neonatal post-exposure prophylaxis and treatment regimens, the paucity of relevant and appropriate neonatal pharmacokinetic and safety data makes their use in neonates and young infants difficult. Twenty-five ARVs have US Food and Drug Administration (FDA) approval for children. Only six are approved for newborn term infants, and one of these drugs (lopinavir) is not recommended until 42 weeks post-conception (See Table 1). Zidovudine, stavudine, lamivudine and emtricitabine have adequate pharmacokinetic data and oral formulations for the first two weeks of life in term infants [11,33,34]. Zidovudine is the only ARV with sufficient pharmacokinetic data from premature infants to allow development of dosing regimens to ensure effective yet not toxic plasma concentrations [35]. For infants unable to tolerate enteral feeding and requiring parenteral drug administration, zidovudine is also the only ARV with an intravenous formulation.

While there are several studies describing nevirapine pharmacokinetics in neonates and young infants, these studies were designed for prophylactic dosing regimens to maintain trough concentrations above 0.1 µg/mL [36]. Nevirapine regimens for successful treatment of HIV infection require trough concentrations above 3.0 µg/mL, but there are no pharmacokinetic studies of nevirapine in neonates with the goal of achieving the treatment concentrations. Nevirapine is metabolized mainly by CYP2B6 and CYP3A4, whose activity is low in term neonates. The nevirapine prophylaxis studies have shown that nevirapine clearance is low in neonates and even lower in preterm or growth-retarded infants [36,37]. In adults, nevirapine auto-induces its own clearance but the extent of auto-induction on immature enzyme systems is unknown. For infants co-infected with *Mycobacterium tuberculosis* and requiring rifampicin co-treatment, nevirapine clearance is also enhanced in African children [38]. Thus, there are fewer antiretroviral options for co-infected newborn infants.

Raltegravir, the first commercially available integrase inhibitor, illustrates many challenges and delays faced in establishing safe and effective ARV dosing regimens for neonates. The FDA approved raltegravir for adults in 2007, for children aged 2 to 12 years in 2011 and for infants four weeks to two years in 2013. However, safety and pharmacokinetic data for neonates are still lacking. Raltegravir is easily transported across the placenta and commonly included in antenatal cART regimens where mothers are on second-line therapy or have not attained virologic suppression towards delivery. Neonatal washout of transplacentally acquired raltegravir is variable and prolonged during the first days of life, as expected since raltegravir is eliminated by UGT1A1, the same enzyme that metabolizes bilirubin and whose activity is very low immediately after birth [39]. An *in vitro* study has shown that at extremely high concentrations (50 to 100 times greater than those seen in adults receiving usual treatment doses), raltegravir displaces bilirubin from albumin [40]. These data suggest that a hyperbilirubinaemic newborn, especially if PTD or LBW, receiving raltegravir could be at increased risk for kernicterus from the displacement of bilirubin from albumin, as was seen with sulfisoxazole [41,42]. Raltegravir dosing for neonates will have to be carefully designed to avoid accumulation to potentially dangerous plasma concentrations, especially when neonates are premature and have LBW. While raltegravir would be of great benefit for use in neonates for prevention or treatment, the rigorous safety and pharmacokinetic studies needed before it can be used safely in term and preterm newborns have yet to be conducted.

Lopinavir-ritonavir is available as a liquid formulation containing 42.4% alcohol by volume and 15.3% propylene glycol by weight and volume. The FDA received a report of 10 neonatal cases of severe renal, metabolic and cardiac toxicities with a single fatality after being given this lopinavir-ritonavir liquid formulation. After this report, a recommendation was issued to avoid lopinavir-ritonavir until two weeks of age and 42 weeks post-conception [43]. It is not known whether these toxicities were caused by excessive exposure to alcohol, propylene glycol and/or lopinavir. However, there are some data to guide dosage in term neonates and premature and LBW infants, should the potential benefit be thought to outweigh the risk [44,45]. The FDA recently approved a lopinavir-ritonavir pellet formulation for children below three years of age for procurement in high prevalence countries. Although it does not contain ethanol or propylene glycol and may be safer for infants, there is concern that newborn infants may not be able to safely swallow the pellets [46].

#### Programmatic and diagnostic challenges in newborn infants

Mothers identified as HIV positive late in pregnancy and at delivery face a very stressful period, learning about their own status and the real possibility that their newborn infants may be HIV infected all in a short space of time. Resources to facilitate counselling in this period are essential. There is growing emphasis on the benefits of breastfeeding HIV-exposed infants in low-income settings and ensuring that mothers continue ARVs in the postpartum period. Feeding and weight monitoring are particularly important in premature and LBW infants. In many hospital settings, flash heating or pasteurization may be used to inactivate HIV in breast milk, while retaining nutritional value [47].

In many settings where HIV is prevalent, newborn infants weighing above 1800 g are already sent home, if stable. Therefore, such LBW infants are seen in primary care facilities such as in Khayelitsha, South Africa, where 28% of HIV-exposed infants delivered were LBW or premature (internal data). Simplified guidelines on PCR testing of HIV-exposed infants (high risk infants versus universal testing for all HIV-exposed infants), indications for up-referral of clinically unwell premature HIV-exposed infants, clear weight-based algorithms on neonatal ART dosages and routine laboratory monitoring all require consideration in primary care settings.

Preliminary data from birth PCR testing of HIV-exposed infants in Khayelitsha indicated a high acceptance of mothers (99%) for testing at delivery. Standardized counselling sessions, including information on repeat testing for neonates negative at birth, were introduced. Counselling sessions were adapted for mother-infant pairs to support cART adherence and appropriate dosing, together with frequent clinical reviews during the first months of life [48]. Neonatal ART is feasible and promotes retention in care when accompanied by good counselling, complemented by disclosure to a treatment supporter [49].

## Conclusions

Neonates including LBW and PTD form a special risk group, requiring increased recognition in the efforts to reach the 90-90-90 targets. Early diagnosis shortly after birth requires planning and resources. There is an urgent need for safe and effective cART regimens. Use of these drugs in this population will require rigorous pharmacokinetic and safety studies and a supportive clinical environment. While several studies through the International Maternal Pediatric and Adolescent AIDS Clinical Trials (IMPAACT) network (P1097, P1106 and P1115) are beginning to address some of the therapeutic gaps in the first weeks of life and in premature infants, these efforts are insufficient. Delays between diagnosis and therapy while waiting for infants to reach a physiological maturity sufficient to allow dosing based on our current knowledge base cannot be justified in term infants and may be dangerous for premature infants.
